# A scoping review of competencies for scientific editors of biomedical journals

**DOI:** 10.1186/s12916-016-0561-2

**Published:** 2016-02-02

**Authors:** James Galipeau, Virginia Barbour, Patricia Baskin, Sally Bell-Syer, Kelly Cobey, Miranda Cumpston, Jon Deeks, Paul Garner, Harriet MacLehose, Larissa Shamseer, Sharon Straus, Peter Tugwell, Elizabeth Wager, Margaret Winker, David Moher

**Affiliations:** Clinical Epidemiology Program, Ottawa Hospital Research Institute, Ottawa, Canada; School of Medicine, Griffith University, Queensland, Australia; American Academy of Neurology, St. Paul, MN USA; Department of Health Sciences, University of York, York, UK; School of Public Health and Preventive Medicine, Monash University, Melbourne, Australia; Institute of Applied Health Research, College of Medical and Dental Sciences, University of Birmingham, Birmingham, UK; Liverpool School of Tropical Medicine, Liverpool, UK; Cochrane Editorial Unit, London, UK; Department of Medicine, University of Toronto, Toronto, Canada; School of Epidemiology, Public Health and Preventive Medicine, Faculty of Medicine, University of Ottawa, Ottawa, Canada; Sideview, Princes Risborough, UK; World Association of Medical Editors, Chicago, USA

**Keywords:** Biomedical, Competencies, Journal, Scientific editor, Scoping review

## Abstract

**Background:**

Biomedical journals are the main route for disseminating the results of health-related research. Despite this, their editors operate largely without formal training or certification. To our knowledge, no body of literature systematically identifying core competencies for scientific editors of biomedical journals exists. Therefore, we aimed to conduct a scoping review to determine what is known on the competency requirements for scientific editors of biomedical journals.

**Methods:**

We searched the MEDLINE®, Cochrane Library, Embase®, CINAHL, PsycINFO, and ERIC databases (from inception to November 2014) and conducted a grey literature search for research and non-research articles with competency-related statements (i.e. competencies, knowledge, skills, behaviors, and tasks) pertaining to the role of scientific editors of peer-reviewed health-related journals. We also conducted an environmental scan, searched the results of a previous environmental scan, and searched the websites of existing networks, major biomedical journal publishers, and organizations that offer resources for editors.

**Results:**

A total of 225 full-text publications were included, 25 of which were research articles. We extracted a total of 1,566 statements possibly related to core competencies for scientific editors of biomedical journals from these publications. We then collated overlapping or duplicate statements which produced a list of 203 unique statements. Finally, we grouped these statements into seven emergent themes: (1) dealing with authors, (2) dealing with peer reviewers, (3) journal publishing, (4) journal promotion, (5) editing, (6) ethics and integrity, and (7) qualities and characteristics of editors.

**Discussion:**

To our knowledge, this scoping review is the first attempt to systematically identify possible competencies of editors. Limitations are that (1) we may not have captured all aspects of a biomedical editor’s work in our searches, (2) removing redundant and overlapping items may have led to the elimination of some nuances between items, (3) restricting to certain databases, and only French and English publications, may have excluded relevant publications, and (4) some statements may not necessarily be competencies.

**Conclusion:**

This scoping review is the first step of a program to develop a minimum set of core competencies for scientific editors of biomedical journals which will be followed by a training needs assessment, a Delphi exercise, and a consensus meeting.

**Electronic supplementary material:**

The online version of this article (doi:10.1186/s12916-016-0561-2) contains supplementary material, which is available to authorized users.

## Background

“*…journals, some of which have reported research for many decades, are still not producing articles that are clear enough to really judge a study’s conduct, quality, and importance—let alone to allow other researchers to reproduce it or build on it*” [1].

Biomedical journals are the main route for disseminating the results of health-related research [2]. However, when examined more closely, the articles that journals publish are problematic; critical details are often missing or poorly reported, consequently reducing their quality, transparency, reproducibility, and usefulness for decision makers [3] – this is wasteful, diminishes scientific and fiscal value, and is unethical [4]. Authors and scientific journals share the majority of the responsibility for these shortcomings, as the former are accountable for the integrity of a study’s conduct and the accuracy of reporting of the content within the manuscript, while the latter are accountable for decisions regarding its publication. On the side of journals, it is scientific editors (by which we mean editors, and ultimately the Editor-in-Chief, who are tasked with making decisions about the content and policies of journals) who are accountable for all material published in their journals. Readers have a right to expect these editors to implement all reasonable actions that could lead to best practices within their journals, as well as journals having processes in place to ensure the quality of the papers they publish.

Unlike many other professional groups, such as clinicians and healthcare professionals, many scientific editors of biomedical journals operate largely without formal training and universal certification is not yet a high priority [5]. Instead, editors generally are invited to serve in their role by publishers, based on their expertise and stature in the field, since such expertise is essential for evaluating research and stature is important for establishing the reputation of the journal and attracting submissions. However, such expertise does not guarantee that editors have the background or training necessary to carry out their roles and responsibilities. Editors may or may not be paid for their role and financial support for the editorial role often does not include travel or training funds. Most editors work part-time as they continue their academic responsibilities in research and/or clinical work, with time for completing editorial responsibilities – much less training – being at a premium. Researchers and peer reviewers similarly have no international standardized formal training or certification as to research conduct, reporting, and evaluation, making the editor’s job even more demanding. This situation is highly problematic given that the consequences of deciding what gets published and the degree of quality that is acceptable impacts future research, decisions, and healthcare directly. Our view is that the lack of consistent training of editors reduces the value of the published literature, including its quality, transparency, and reproducibility, thereby reducing value for money to funders and the usability of research findings, ultimately degrading public trust in the research record [3]. However, we are unaware of any research that directly addresses this topic. Additionally, while the training of biomedical editors is an important mechanism to ensure the quality of the published literature, other important changes in tandem with this, including re-examining the training offered to peer-reviewers and training graduate students in study conduct, analysis, interpretation, and reporting, could also have a beneficial effect.

Some organizations, for example, the World Association of Medical Editors (WAME) [6] and the Committee on Publication Ethics (COPE) [7], provide rigorously-developed resources for biomedical journal editors free of charge, including guidance on the role of the medical editor, editorial policies, and listservs on which editors’ questions and issues are discussed. There are also individual websites and blogs, such as “Journalology” [8], that provide thoughtful commentary on current issues related to publication science. Several commercial groups offer short training courses for editors [9, 10]. However, for any comprehensive editor training program to work effectively and be assimilated into practice, it must be based on what the broader biomedical editor community considers to be core competencies.

We are unaware of any body of literature systematically identifying core competencies for biomedical editors, nor any agreement on or attempt at a consensus process to determine what they should be. For the purposes of this research, we borrow from the literature on competency-based continuing professional development to define competence as “*the array of abilities across multiple domains or aspects of* [practitioner] *performance in a certain context*” [11]. We thus define core competencies as the essential knowledge, skills, and behaviors necessary for the practice of scientific editing of biomedical journals. We believe it is important to develop a set of core competencies so that training programs can then be developed and tailored with the intent that all editors meet some basic globally agreed-upon standards. Other stakeholders, such as publishers (including medical associations who have their own journals), peer reviewers, and authors (researchers), also need to contribute to this effort. Herein, as a starting point, we report a scoping review of possible core competencies of scientific editors of biomedical journals.

### Objectives

The objective of this scoping review was to conduct a systematic search of the literature on the competencies required for scientific editors of biomedical journals to effectively and efficiently produce transparently reported and correctly analyzed and interpreted publications. Our specific aim was to answer the research question: “What is known from the literature on the competency requirements of scientific editors of peer reviewed biomedical journals?” with the goal of summarizing the existing literature. The purpose of this scoping review is to inform the future development of a set of core competencies for scientific editors of biomedical journals, which we hypothesize will ultimately lead to improvements in the quality of the published literature.

## Methods

The protocol for this project has previously been deposited in the University of Ottawa’s Digital Repository (uOttawa Research) prior to beginning the screening phase [12]. Our methodological approach was guided by the Arksey and O’Malley Framework [13], as well as the additional suggestions to this framework made by Levac [14]. Specifically, we undertook the six-step process of: (1) identifying the research question; (2) identifying relevant studies; (3) study selection; (4) charting the data; (5) collating, summarizing, and reporting results; and (6) consultation.

### Search strategy

We searched the MEDLINE®, Cochrane Library, Embase®, CINAHL, PsycINFO, and ERIC databases, all from inception to November 10th, 2014 (Additional file 1). The specific search strategies were created by a Health Sciences Librarian with expertise in scoping review searching. The MEDLINE strategy was developed with input from the project team, then peer reviewed by a second librarian not otherwise associated with the project using the PRESS standard [15]. We also conducted a grey literature search, which included hand searching the reference lists of included articles, as well as searching key journals, in particular *JAMA* from 1989, and *BMJ*, *PLoS Medicine*, *European Science Editing*, *Annals of Internal Medicine*, and *CMAJ* from 2004 onward to identify publications related to the Peer Review Congresses [16].

#### Expanded scoping exercise

Given that this scoping review is part of a larger program to develop core competencies for scientific editors of biomedical journals, in addition to seeking research literature, we also incorporated an expanded scoping exercise that included non-research-based (published and unpublished) materials to fill an expected gap in research on competencies of scientific editors of biomedical journals. This expectation was based on a previous systematic review [17], which found no comparative studies on training for scientific editors of biomedical journals, and an associated environmental scan that found only a few training opportunities for these editors [18]. Additionally, our anecdotal experience was that most descriptions of editor competencies are found in editorial-type publications (e.g. commentaries), job postings, and guidance documents (e.g. COPE Guidelines for Editors) – all of which would not generally be captured in a traditional scoping review of published research.

The expanded scoping exercise included (1) searching the databases from the search strategy and grey literature for non-research-based publications, (2) searching the results of an environmental scan from a previous related project [17, 18], (3) conducting a new environmental scan with additional search terms, and (4) searching the websites of existing networks (i.e. EQUATOR Network), major biomedical journal publishers (i.e. Wiley-Blackwell, Elsevier, BioMed Central, BMJ Publishing Group, Springer), and organizations that offer resources for editors (i.e. COPE, WAME, Council of Science Editors, European Association of Science Editors, and International Committee of Medical Journal Editors). For the database searches, the full-text of all potentially relevant documents were retrieved and independently reviewed for eligibility, in duplicate, by members of the team using a priori eligibility criteria. Disagreements between reviewers were resolved by consensus or by a third member of the research team. Both environmental scans employed the same methodology, which involved the use of the Google search engine to run a series of two- and three-word keyword searches (Additional file 1). For each set of search outcomes, the first 50 Google results were screened for relevant information. If any of the last 10 results contained useful information, another 10 results were screened. This process was continued until a set of 10 results with no relevant information was found [18].

### Study selection: inclusion criteria

#### Population

Articles with statements mentioning competencies, knowledge, skills, behaviors, and tasks (henceforth referred to as ‘competency-related statements’) pertaining to the role of scientific editors of peer-reviewed health-related journals (including Editors-in-Chief and associate/academic editors, and full-time professional journal editors) were included. Articles related to all other types of editor not directly involved in evaluation, peer review, revision, and selection of content (e.g. managing editors, technical editors, copy editors) were excluded.

#### Disciplines

We adopted MEDLINE’s journal selection criteria for our definition of health [19]. This definition includes journals that are “*predominantly devoted to reporting original investigations in the biomedical and health sciences, including research in the basic sciences; clinical trials of therapeutic agents; effectiveness of diagnostic or therapeutic techniques; or studies relating to the behavioral, epidemiological, or educational aspects of medicine*” [19]. This definition encompasses biomedical journals as well as those in the disciplines of psychology and education. For feasibility purposes, we did not include journals from the physical or natural sciences.

#### Study designs

The review included all study designs as well as editorials and commentaries. Economic evaluations and letters were excluded, as neither was expected to contribute useful data for the purposes of this scoping review. For feasibility purposes, we included articles written in English and French only. We did not include English or French abstracts of papers written in another language.

### Screening

Following the execution of the search strategy, the identified records (titles and available abstracts) were collated in a Reference Manager [20] database for de-duplication. The final unique record set of potentially eligible studies was exported to Internet-based software, DistillerSR (Evidence Partners, Ottawa, Canada), through which screening of records and data extraction were carried out. Each title and abstract was screened by two of four reviewers (LS, JG, JT (see Acknowledgements), and MG (see Acknowledgements)) using a ‘liberal accelerated’ method [21] (i.e. one reviewer screened each record and a second reviewer screened only excluded records, independently). The full-text of all remaining potentially eligible papers was then retrieved, uploaded into DistillerSR, and reviewed for eligibility, independently, by two members of the team (LS and JG) using a priori eligibility criteria. Disagreements between reviewers at this stage were resolved by consensus or by a third member of the research team.

### Charting the data

A data extraction form was developed a priori to capture information on each document included in the review. It was piloted and refined based on feedback from the exercise. Three people (JG, KDC, LS) carried out data extraction in the following manner: data were extracted by one reviewer and a second reviewer conducted verification of the data for all records. Disagreements between reviewers were resolved by consensus. General study characteristics extracted for the database search were: first author name and contact information (of corresponding author), year of publication, institutional affiliation of first author, country, language of publication, study design, and funding source. For the environmental scans we extracted the URL, title of the document, language of publication, and who produced the document (affiliation). For all documents, we collected descriptions of any statements potentially relating to the competencies of scientific editors of biomedical journals, such as descriptions of particular skills, knowledge, attitudes, behaviors, tasks, and training.

### Collating, summarizing, and reporting the results

In an effort to create a useful summary of the data for the next steps of our program to develop core competencies for scientific editors of biomedical journals, we combined the competency-related statements retrieved from all sources. First, two people (JG, KDC) classified all statements pertaining only to Editors-in-Chief into a single category, since these would be considered to be beyond the core competencies of scientific editors more generally. They then collated overlapping or duplicate statements to produce a list of unique statements. Finally, they grouped statements into emergent themes to make them more manageable for future use (e.g. in an upcoming Delphi exercise), and so that they would be understandable to readers. While some of the wording of particular statements was modified to assimilate overlapping statements, where statements were expressed as knowledge, skills, behaviors, or tasks that implied competencies, but not as competencies themselves, we did not edit or translate them to express competencies, in order to preserve the original intent. The relationships between behaviors, tasks, and competencies will be the subject of discussion and translation undertaken as part of the consensus meeting phase of this project.

## Results

### Database search

**Fig. 1:**
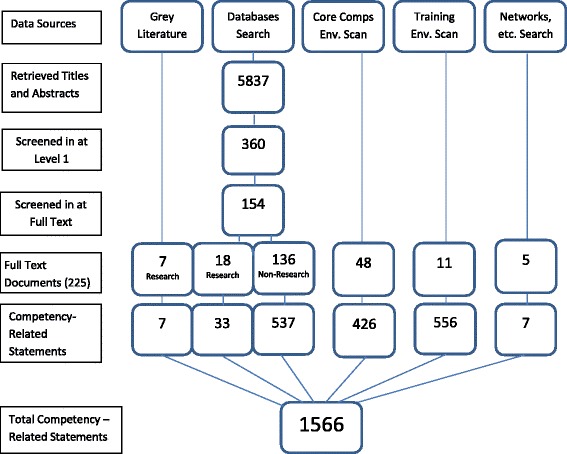
Study flow diagram

We screened 5,837 titles and abstracts, of which 360 were screened in full-text (Fig. 1). Of these, 206 were excluded, leaving 154 publications meeting the inclusion criteria. Twenty-five of these publications were research based (Table 1) and the remaining 136 were editorial in nature (Additional file 2).

**Table 1:** Included research-based publications

First author	Affiliation	Country	Journal	Year	Design	# ^a^	Item(s) ^b^
Albert, T	Tim Albert Training	UK	Learned Publishing	2002	Survey	0	^N/A^
Barnes, M	University of Nebraska-Lincoln	USA	The Review of Higher Education	1986	Survey	5	^5, 72, 170, 171, 185^
Carroll-Johnson, R	Oncology Nursing Society	USA	Nurse Author & Editor	1996	Survey	1	^137^
Davis, RM	Henry Ford Health System	USA	Science & Engineering Ethics	2002	Survey	1	^137^
de Jesus Mari, J	King’s College, University of London	UK	African Journal of Psychiatry	2009	Task Force Report	0	^N/A^
Etemadi, A	Shaheed Beheshti University of Medical Sciences	Iran	Saudi Medical Journal	2004	Survey	1	^141^
Freda, M	Journal of Nursing Scholarship	USA	Journal of Nursing Scholarship	2005	Survey	0	^N/A^
Froehle, T	Indiana University, Bloomington	USA	Counselor Education & Supervision	1990	Descriptive Study	0	^N/A^
Galipeau, J	Ottawa Hospital Research Institute	Canada	Systematic Reviews	2013	Systematic Review	0	^N/A^
Garrow, J	LOCKNET Peer Review Research Group: European Journal of Clinical Nutrition	UK	Journal of the American Medical Association	1998	Survey	0	^N/A^
Grindlay, D	Centre for Evidence-based Veterinary Medicine, School of Veterinary Medicine and Science, The University of Nottingham	UK	BMC Veterinary Research	2014	Survey	1	^148^
Hing, C	Department of Trauma & Orthopaedics, St George’s Hospital, Tooting, UK	UK	Journal of Orthopaedic Surgery & Research	2011	Survey	0	^N/A^
Kearney, M	University of Rochester School of Nursing	USA	Nursing Outlook	2006	Descriptive Study	13	^5, 26, 30, 35, 69, 72, 83, 84. 85, 91, 102, 178, 203^
Kleinert, S	The Lancet	UK	Peer Review Congress (Abstract)	2005	Observational Study	0	^N/A^
Lebeau, DL	Tulane University Medical Center	USA	Peer Review Congress (Abstract)	1997	Survey	1	^72^
Logothetti, H	Obstetrics & Gynecology	USA	Peer Review Congress (Abstract)	2009	Case-Control	1	^101^
Patrone, D	Philosophy Department, State University of New York at Oneonta/Broome Community College, Binghamton, New York	USA	Biosecurity and Bioterrorism: Biodefense Strategy, Practice, and Science	2012	Survey	2	^147, 203^
Radford, D	Division of Prosthetic Dentistry, King’s and St Thomas’ Dental Institute, London	UK	British Dental Journal	1999	Survey	0	^N/A^
Reynolds, T	Highland Hospital	USA	Peer Review Congress (Abstract)	2009	Survey	0	^N/A^
Silverman, R	Ohio State University	USA	None (Final Report)	1975	Final Report	6	^79, 91, 101, 166, 194, 203^
Srinivasan, S	Indian J Medical Ethics	India	Peer Review Congress (Abstract)	2013	Survey	0	^N/A^
Wager, E	Sideview, Princes Risborough	UK	Peer Review Congress (Abstract)	2009	Case Analysis	0	^N/A^
Wager, E	Sideview, Princes Risborough	UK	The British Medical Journal	2013	Quantitative + Interviews	1	^136^
Williams, P	University College London	UK	Science and Engineering Ethics	2011	Case Studies	2	^58, 203^
Wong, V	Department of Neurology, University of Michigan, Ann Arbor	USA	Journal of Clinical Epidemiology	2011	Survey	5	^2, 118, 123, 203(2)^
TOTAL						40	

^a^ Number of competency statements extracted from the document

^b^ Corresponds to the item number from the list of competency-related statements (Table 3)

### Charting the data

#### General characteristics

##### Research-based publications

A total of 18 publications from the database search presenting the results of research (subsequently termed ‘research-based publications’) were considered relevant to core competencies for scientific editors of biomedical journals, along with another seven articles found in the grey literature search (including six conference abstracts for which there appears to be no full text publication) (Table 1). None of these 25 articles had an outline or description of core competencies of scientific editors as an objective of the research. Fifteen studies were survey-based research, three were descriptive studies, three were case studies, and two were final project reports (one from a task force and the other from a study funded by the US National Institute of Education). The remaining two studies were a systematic review and a mixed-methods study. Five studies reported receiving funding. Nineteen studies were published in different journals and the remaining six were part of the Peer Review Congress Proceedings. Publication dates ranged from 1975 to 2014; 2009 was the year with the most publications (n = 3), followed by 2011 (n = 2) and 2013 (n = 2). Twelve of the studies’ first authors were from the USA, 10 from the United Kingdom, and one each from Iran, India, and Canada. The publications produced a total of 40 competency-related statements (i.e. possible competencies), with individual publications yielding between zero and 14 statements and a median of one statement per publication.

#### Expanded scoping exercise

##### Non research-based publications

A total of 136 non-research-based publications were considered relevant to core competencies for scientific editors of biomedical journals, yielding a total of 537 competency-related statements (Additional file 2). Similar to the research-based literature, none of these publications had the explicit goal of outlining a set of core competencies for scientific editors. Overall, 133 publications were editorial in nature, while the remaining three included a lecture, a job description, and an interview. Seventeen journals had multiple included publications, with three of them (*Australian and New Zealand Journal of Obstetrics and Gynaecology*, *Croatian Medical Journal*, and *Indian Journal of Medical Sciences*) producing three publications each while the other 14 journals had two publications each. *JAMA* also had four publications across three journals in its collection (*JAMA*, *JAMA Internal Medicine*, *JAMA Ophthalmology*). The rest of the sample consisted of a single publication per journal. The date of publication ranged from 1954 to 2015; 2011 (n = 20) and 2012 (n = 17) were the 2 years with the most studies. The sample included 66 studies with first authors originating from the USA and 19 from the United Kingdom, with representation from another 18 countries as well among first authors. The individual publications yielded between zero and 15 competency-related statements, with a median of two statements per publication.

##### Environmental scan of training in Journalology

We reviewed all 258 documents listed in the Repository of Ongoing Training Opportunities in Journalology [20], which houses all of the data from an environmental scan of training in Journalology carried out by members of our team in 2013. From this repository, we extracted 11 relevant non-research-based articles from which we were able to retrieve 556 competency-related statements relating to scientific editors of biomedical journals (Table 2). Seven of these documents were from organizations that provide guidance to editors (e.g. WAME, COPE, International Committee of Medical Journal Editors) and the remaining documents were from a variety of other sources.

**Table 2:** Included documents from expanded scoping exercise

GUIDANCE FROM EDITORIAL GROUPS				
Title	Editorial group/organization	Scanning source	# ^a^	Item(s) ^b^
International Standards for Authors	Committee on Publication Ethics (COPE)	Training	58	^6(2), 9, 10, 11, 12(2), 20(6), 22(3), 25, 26, 34, 35(3), 39, 46, 57(2), 64, 72(5), 79(2), 108(2), 137(2), 138(4), 140, 141(4), 143, 144, 145, 146(3), 150, 159, 198, 199, 203^
Guidelines for Editors	COPE	Training	69	^2, 6, 16, 19, 20, 21,22(2), 24, 25, 26(2), 27, 28, 30(2), 32, 34, 35(4), 39, 41, 42, 46, 54, 61, 67, 72(6), 79(2), 80, 83, 87(2), 88, 104, 111, 137(2), 138, 139, 141(2), 143, 146, 150, 153(2), 159, 189, 194, 197, 198, 199(2), 203(7)^
A Short Guide to Ethical Editing for New Editors	COPE	Training	34	^6(2), 10, 20(3), 22, 26(2), 30, 32, 35(2), 36, 48(2), 62, 68, 72, 104, 116, 136, 137(2), 138, 144(2), 189, 197, 203(5)^
A Science Editing Course for Graduate Students	Council of Science Editors (CSE)	Core competencies	6	^5, 95, 98, 135, 138, 178^
Can Non-Native-English-Speaking Editors be Effective Editors of English-Language Writing?	CSE	Core competencies	13	^25, 78, 95, 100(2), 126(2), 127, 132, 138, 141, 178, 199^
Guidelines for Reporting Health Research: How to Promote their Use in Your Journal	EQUATOR Network	Networks search	0	^N/A^
Research Ethics, Publication Ethics and Good Practice Guidelines	EQUATOR Network	Networks search	0	^N/A^
European Association of Science Editors (EASE) Toolkit for Journal Editors	EASE	Core competencies	0	^N/A^
Editor’s Handbook (2nd Edition)	EASE	Core competencies	166	^2(2), 7, 8, 9, 10, 15(2), 16, 18, 20(2), 22(4), 24(2), 25, 26(2), 27(2), 28, 30, 31, 32, 33, 35(3), 36, 39(2), 48, 50(3), 52(2), 54(3), 57(3), 58, 59, 61, 63, 64, 65(6), 69(2), 70, 71, 72(2), 73(2), 77, 78, 79(2), 83(2), 84, 85(3), 87, 89(2), 91(2), 92, 93, 96(2), 98, 105, 108(2), 109(3), 111, 113, 116, 117(4), 124(3), 125(15), 127, 131(2), 133(2), 135(2), 137(2), 138(2), 139(4), 141(3), 142(3), 144, 148(2), 153(3), 158, 159, 167, 171, 194, 197, 199(2), 200(2), 201(3), 202(2), 203(8)^
Golden Rules for Scholarly Journal Editors	EASE	Core competencies	10	^6, 15, 35, 72(2), 85, 89, 111, 138, 149^
Recommendations for the Conduct, Reporting, Editing, and Publication of Scholarly Work in Medical Journals	International Committee of Medical Journal Editors (ICMJE)	Core competencies	2	^35, 140^
Responsibilities in the Submission and Peer Review Process (Journals)	ICMJE	Training	9	^1, 13, 38, 46, 47(2), 140(3)^
Syllabus for Prospective and Newly Appointed Editors	World Association of Medical Editors (WAME)	Training	56	^6, 10, 14, 18, 20(4), 26(3), 29, 35, 43, 47, 48(2), 52, 57(6), 61(2), 62, 72(2), 79, 83, 84, 91, 92, 106, 113, 119, 137, 139, 141(2), 146(2), 159, 189, 197(2), 203(9)^
Outline of Planned WAME Journal Editor Training	WAME	Training	84	^17, 20(2), 22, 26(5), 33, 37, 48, 50, 51, 52, 56(2), 59, 61, 64(3), 65(3), 66, 68(5), 70, 72, 74, 75, 76, 80, 81, 84, 90, 91(2), 106, 109, 113(4), 124(3), 127, 128, 132, 133(2), 135(2), 136, 137, 140, 141(5), 142, 144, 145, 146, 152, 154, 155(3), 159, 162(2), 166, 198, 203(4)^
ASSOCIATIONS, JOURNALS, PUBLISHERS				
Editor Handbook	Alliance of Crop, Soil, and Environmental Sciences Society	Core competencies	0	^N/A^
Editors and Reviewers	Alliance of Crop, Soil, and Environmental Sciences Society	Core competencies	11	^6(3), 35(2), 47, 61, 112(2), 140, 141^
Editor Handbook	American Association of Pharmaceutical Scientists Journal	Core competencies	9	^35, 91, 116(2), 203(5)^
Editor-In-Chief: Position Description	American Geophysical Union	Core competencies	25	^69, 72, 84, 95, 140, 159, 160, 161, 162(2), 166, 167, 170(2), 171(2), 178, 181(2), 182, 189, 191(2), 199, 203^
Position Description for the AJNR Editor and Basic Qualifications	American Journal of Neuroradiology	Core competencies	5	^5, 70, 91, 117, 168^
Responsibilities of an Editor	Annals, Academy of Medicine, Singapore	Training	30	^6(2), 17, 19, 35(2), 61, 73, 77, 79, 80, 81, 83, 90(4), 91, 92, 137(4), 138, 151, 172, 192, 203(3)^
What does an Associate Editor Actually do?	Association for Computing Machinery	Core competencies	3	^34, 40, 72^
The Role of the Scientific Editor	British Dental Journal	Core competencies	3	^8, 14, 20^
Recruiting a Journal Editor: An HSS Challenge	Cambridge Journals Blog	Core competencies	0	^N/A^
Editorial: on Editing and Being an Editor	Cultural Studies of Science Education	Core competencies	3	^6, 33, 34^
Editor’s Pack	Elsevier	Publishers search	0	^N/A^
How do Publishers Choose Editors, and How do they Work Together?	Elsevier	Core competencies	7	^110, 160, 162, 184, 185, 194, 203^
Publishing Ethics Resource Kit (PERK)	Elsevier	Publisher search	0	^N/A^
European Respiratory Journal Editor(s)-in-Chief	European Respiratory Society	Core competencies	6	^162(2), 165, 167, 195, 178^
Editor-in-Chief (position description)	International Society of Exposure Science; Journal of Exposure Science and Environmental Epidemiology	Core competencies	9	^19, 35, 101, 138, 159, 165, 166, 170, 203^
Editor-in-Chief (position description)	Journal of Family Planning and Reproductive Health Care	Core competencies	19	^8, 19(2), 36, 95, 116, 117, 121, 123(2), 127, 138, 159, 164, 167, 170, 178, 180, 181^
Responsibilities of the Editor	Journal of Medical Internet Research	Core competencies	1	^123^
Responsibilities of the JNCI Editor‐in‐Chief	Journal of the National Cancer Institute	Core competencies	8	^95, 99, 127, 132, 138, 159, 174, 203^
Responsibilities of Editors and Reviewers	Online Ethics Center for Engineering and Science	Core competencies	0	^N/A^
Scientific Editing--A Wise Career Choice	Science Careers (from the journal Science)	Core competencies	0	^N/A^
Horses for Courses--Research Papers versus Reviews	Science Careers (from the journal Science)	Core competencies	0	^N/A^
The Editors' World: Back to the Books	Science Careers (from the journal Science)	Core competencies	6	^159(2), 165, 175, 178, 181^
Bench to Page: An Editor's View of Science Publishing	Science Careers (from the journal Science)	Core competencies	3	^73, 164, 170^
At the Gateway of Cutting-Edge Research	Science Careers (from the journal Science)	Core competencies	7	^68, 108, 109, 122, 162, 163, 191^
Translating Scientific Expertise into Publishing Success	Science Careers (from the journal Science)	Core competencies	0	^N/A^
Journal Editors Get Twitter-Savvy	Science Careers (from the journal Science)	Core competencies	14	^40, 71, 73, 90, 93, 95, 108, 164, 165, 174, 177, 185, 193, 196^
Careers in Neuroscience/Career Paths: Science Publishing	Society for Neuroscience	Core competencies	8	^122, 164, 165, 166, 167(2), 178, 184^
Academic Journal Editors’ Professionalism: Perceptions of Power, Proficiency and Personal Agendas	Society for Research Into Higher Education	Core competencies	9	^33(2), 72, 73, 110, 121, 160, 167, 174^
Editorial Guide	Springer	Publishers search	7	^26, 65, 78, 82, 85, 138, 197^
Trainee Programs/Editorial Trainees	Springer	Core competencies	0	^N/A^
Confessions of a Journal Editor	The Chronicles of Higher Education	Core competencies	0	^N/A^
Ethics and the Psychiatry Journal Editor: Responsibilities and Dilemmas	The Israel Journal of Psychiatry and Related Sciences	Core competencies	9	^20(2), 22, 36, 48, 64, 120, 140, 141^
Editor Ethics 2.0 Code/Affirming Editors	University of North Carolina – Charlotte	Core competencies	2	^20, 138^
UOJM Editor Training: Results from the 2013 Editor Satisfaction Survey and Highlights from 2013–2014 Training Workshops	University of Ottawa Journal of Medicine	Core competencies	8	^36, 61, 74, 79, 95, 123, 160, 175^
FAME Guidelines	World Health Organization	Training	35	^6(3), 10, 14, 15, 30, 34, 35, 39, 40(2), 47, 61, 72(3), 75, 79(2), 91, 92, 133, 138(2), 141, 143, 149, 159(2), 179, 189, 198, 203(2)^
Research Journal Editor Position Description	Young Adult Library Service Association	Core competencies	11	^35, 95(2), 117, 159, 160, 161, 162, 167, 174, 180,^
OTHER SOURCES				
Medical/Scientific Editor (job posting)	Alexion Pharmaceuticals, Inc.	Core competencies	0	^N/A^
Duties of Editors	Bioinfo Publications	Core competencies	3	^104, 141, 199^
What is Special about Science Editing?	Biotext	Core competencies	11	^120, 124, 125, 129(2), 130, 131, 141, 165, 175, 193^
What is Different about Science Editing?	Emend Editing	Core competencies	13	^57, 60, 117, 125, 126, 127, 129, 130, 131, 162, 165, 175, 193^
What Exactly Does an Editor Do?	Joseph Alpert	Training	11	^39, 54, 68, 73, 86, 108, 138, 162, 198, 203(2)^
Becoming a Journal Editor	PhD2Published	Core competencies	5	^109, 167, 182, 190, 199^
Public Knowledge Project School	Public Knowledge Project	Training	67	^6(3), 14, 15(8), 20, 26(3), 30, 34, 35(3), 39, 46, 47, 50, 53(3), 57(2), 59, 61(2), 62, 68, 84, 85, 87, 91, 95, 104(2), 137(2), 138, 140, 141, 149, 160, 167(2), 197(2), 203(14)^
What does an Editor (a Member of Editorial Board) do Exactly in Journals?	ResearchGate	Core competencies	0	^N/A^
What are the Role and Duties of a Scientific Editor of an Academic Peer-Review Journal?	ResearchGate	Core competencies	5	^44, 72, 79, 159, 203^
Job Description of an Editor-in-Chief	Study.com	Core competencies	0	^N/A^
So, you want to be a Science Writer when you grow up…	The Black Hole	Core competencies	0	^N/A^
Ideas for a Topical Outline	Unknown	Training	103	^2, 8(2), 10, 14, 15, 17, 20, 22, 26(3), 31, 33, 37, 48, 49, 50, 51, 52, 53, 56(2), 57, 59(2), 61(2), 62, 64(3), 66, 68(4), 70, 71, 72, 74, 75, 76, 77(2), 81, 82, 91(3), 92, 95, 101, 106(2), 113(3), 117, 124(3), 127, 133(4), 135(2), 136, 137, 140, 141(3), 142, 144, 145, 146, 147, 152, 154, 155(3), 161, 162(2), 165(2), 169(2), 173, 198, 203(9)^
Recommended Recruitment Steps for Journal Editor, CJNSE/RCJCÉ	Wired Learning Consultants	Core competencies	6	^95, 117, 162, 167, 178(2)^
How do I Become a Science Editor?	WiseGEEK	Core competencies	0	^N/A^
TOTAL			989	

^a^ Number of competency statements extracted from the document

^b^ Corresponds to the item number from the list of competency-related statements (Table 3)

##### Environmental scan of core competencies for scientific editors of biomedical journals

The environmental scan carried out for this project consisted of a total of 40 keyword searches (Additional file 1) that yielded 48 relevant documents, of which 35 were deemed to meet the eligibility criteria after screening in duplicate. These 35 documents produced 426 competency-related statements (Table 2). Among the sample, 18 documents were produced or published by journals, nine were from associations and societies, six were from organizations providing guidance to editors, and two were from publishers.

##### Search of networks, publishers, and meetings

A search of networks, publishers, and meetings produced an additional five documents, three of which were documents from publishers’ websites and two from the EQUATOR Network (Table 2). We extracted seven competency-related statements from these additional documents.

Of the 64 documents in the expanded scoping exercise, the European Association of Science Editors Editor’s Handbook (2nd Edition) [22] had the highest number of competency-related statements (n = 166). The median number of competency-related statements across the scoping exercise documents was six.

### Collating and summarizing the data

The combined 1,566 competency-related statements originating from the 225 total documents were collated and then de-duplicated, producing a list of 203 unique statements (i.e. possible competencies of scientific editors) (Table 3). The statements were organized into seven categories that emerged from the data: (1) dealing with authors; (2) dealing with peer reviewers; (3) journal publishing; (4) journal promotion; (5) editing; (6) ethics and integrity; and (7) qualities and characteristics of editors.

**Table 3:** Competency-related statements ^a^

Item # ^b^	Competency-related statement	# ^c^
I. Dealing with authors		
Scientific editors should:		
1	Review study protocols and methods and encourage authors to make them publicly available	1
2	Ensure authors are aware of ethical authorship practices	5
3	Seek to help authors understand magnitude of effect	1
4	Assist potential authors in developing a spirit of inquiry	1
5	Develop wide acquaintance with potential authors	4
6	Demonstrate accountability to authors and ensure they are treated with fairness, courtesy, and objectivity	36
7	Provide constructive criticism to authors	3
8	Engage in mentorship and education of authors to help them produce work to best effect	30
9	Mediate sound communication between the comments of reviewers and responses of authors	4
10	Ensure publication decisions are clearly communicated to all authors	7
11	Interact with authors to confirm undisputed changes in authorship and act on any institutional findings concerning authorship disputes	1
12	Clarify the peer-review processes to authors	2
13	Negotiate manuscript publication delays with authors	1
14	Deal with authors who appeal against rejection	7
15	Ensure authors are informed about journal and article information and/or funding	13
16	Ensure that requests from authors that an individual not review their submission are respected, if these are well-reasoned	2
17	Engage in critical evaluation of authors’ manuscripts and the peer-review process itself	3
18	Provide active encouragement for revisions of manuscripts	2
19	Demonstrate experience as a competent author, academic, researcher, or reviewer	6
20	Demonstrate proficiency in dealing with author misconduct and other issues related to publication ethics	35
21	Work with publishers to defend author rights and pursue offenders	1
22	Act on concerns about plagiarism, data fabrication, or an authorship issue and follow-up with authors and then institutions	13
23	Request full disclosure of potential conflicts of interest by the authors	2
24	Support authors in dealing with breaches of copyright and plagiarism issues	4
25	Request appropriate documentation from authors when they submit manuscripts	6
II. Dealing with peer reviewers		
26	Develop, facilitate, and monitor the peer review process	32
27	Knowledge of different types of peer review	4
28	Encourage and demonstrate awareness of new findings on peer review and publishing and how these influence their journal’s processes	3
29	Review revised manuscripts	1
30	Provide guidance to peer reviewers	11
31	Ensure thorough statistical review	3
32	Ensure that peer review panels for individual papers are not biased	4
33	Evaluate and provide feedback to the reviewers on review quality	9
34	Ensure manuscript content is matched with the expertise of particular reviewers	13
35	Monitor and ensure the fairness, timeliness, thoroughness, and civility in the processing of manuscripts and in responding to queries from authors and reviewers	37
36	Demonstrate knowledge of the workings of the peer review process	7
37	Train peer reviewers	2
38	Ensure reviewer comments are shared with all peer reviewers	1
39	Synthesize reviews and make ultimate editorial decisions in light of peer reviewers’ comments	10
40	Evaluate manuscripts in light of reviewers’ critiques and various selection criteria	5
41	Demonstrate the ability to distinguish between objective peer reviewed research and reviews from opinion and the journal content from advertising and other promotional content	2
42	Ensure reviewers who consistently produce discourteous, poor quality, or late reviews are removed from the journal’s pool of peer reviewers	1
43	Ensure a decision is made on a manuscript when reviewers fail to submit a timely review	1
44	Ensure a very high standard of the referees, don’t accept sloppy reports from anyone	2
45	Demonstrate publication and reviewing skills and experience	1
46	Ensure that reviewers keep manuscripts, associated material, and the information they contain strictly confidential	6
47	Demonstrate sound judgment in the acceptance of research articles, editorials, and reviews that touch on current issues	7
III. Journal publishing		
48	Demonstrate knowledge of marketing and advertising policies, including ethical issues	9
49	Demonstrate knowledge of the article embargo process	1
50	Demonstrate knowledge of indexing services	7
51	Demonstrate knowledge of reprint processes	2
52	Demonstrate knowledge of the specifications of the journal	5
53	Demonstrate knowledge of the goals of the journal	4
54	Demonstrate knowledge of formatting of layout for journal issues	6
55	Ensure the content of manuscripts submitted for publication is checked for accuracy	1
56	Demonstrate knowledge of the different parts, purposes, and characteristics of different types of journals	4
57	Demonstrate understanding of the editorial office and operations	22
58	Ensure that selected/published research is correct	2
59	Demonstrate knowledge about legal issues relating to the position of scientific editor	5
60	Be aware of how design can be used to improve the readability of a document	1
61	Demonstrate understanding of one’s responsibilities and rights as a journal editor	14
62	Demonstrate knowledge of the roles and responsibilities of the editorial staff	4
63	Identify and address issues related to data protection and confidentiality	2
64	Demonstrate knowledge of journal metrics and research impact	9
65	Demonstrate knowledge of online publishing and products	13
66	Demonstrate knowledge of the parts, purposes, and characteristics of audio and video clips	2
67	Demonstrate awareness of intellectual property issues and work with publisher to handle potential breaches	1
68	Demonstrate knowledge of technical-economical aspects of medical journal production	16
69	Explore and embrace innovative technologies	5
70	Maintain close contact with the latest trends in electronic media (e.g. tablets)	4
71	Engage in multimedia publishing practices	3
72	Act as a gatekeeper and guarantor of publications, checking both the quality and scope of research published in the journal	81
IV. Journal promotion		
73	Maintain knowledge of important developments and trends in one’s own field	10
74	Demonstrate knowledge of history of journals and scientific publications	2
75	Demonstrate knowledge of national and regional variations between journals	3
76	Demonstrate knowledge of political and geopolitical issues	2
77	Demonstrate familiarity with associations and their educational resources	5
78	Stay on top of updates in one’s field	3
79	Demonstrate knowledge of, and work to maintain and improve the journal’s policies, vision, scope, content, processes, and goals	20
80	Ensure decisions are based on the validity of the work and its importance to the journal’s readers	4
81	Ensure controversial topics (political, ethical) are dealt with	3
82	Stimulate others to write articles and editorials	3
83	Engage in the promotion of scholarly research and best practices in conducting and reporting it	9
84	Entice leading researchers to submit to the journal	7
85	Serve as ambassador for the journal in establishing its visibility and image	10
86	Motivate physicians to read, ponder, and implement the information provided	1
87	Seek feedback/opinions on the journal	4
88	Enhance public understanding of science	3
89	Demonstrate understanding of who one’s constituency is	44
90	Demonstrate a responsibility to the scientific community	8
91	Hold paramount the interests of the particular journal’s readers	26
92	Engage in communication with the public	8
93	Engage with existing and new scientific communities	2
V. Editing		
94	Demonstrate knowledge of policies for submission of manuscripts	1
95	Demonstrate broad and detailed knowledge of the skills needed to refine a piece of scientific work and shepherd it through to publication	27
96	Demonstrate knowledge of typography	4
97	Demonstrate knowledge of and experience with online editing	1
98	Demonstrate knowledge of the fundamentals of editing various types of science copy	3
99	Enforce ICMJE authorship guidelines	2
100	Ensure logic and consistency of manuscripts	2
101	Demonstrate the ability to assess the quality of papers	7
102	Ensure papers selected are clinically relevant	3
103	Ensure papers selected have a clear story-line	1
104	Demonstrate the ability to select material for its merit, interest to readers, and originality alone	11
105	Ensure papers selected are suitable to the journal	4
106	Ensure papers selected for review are meaningful	5
107	Ensure manuscripts are triaged judiciously (for journals that use such a process)	2
108	Demonstrate the ability to form preliminary opinions on a submitted manuscript’s relevance	8
109	Demonstrate the ability to make fast, good decisions about papers	6
110	Demonstrate the ability to make difficult decisions	5
111	Demonstrate the ability to exercise excellent judgment	8
112	Handle manuscripts in the areas of one’s expertise and assist in finding persons qualified to handle papers in those areas outside one’s expertise	2
113	Engage in and maintain interactions and good relations with media	11
114	Select, curate, and comment on articles for publication	1
115	Ensure alterations recommended based on peer reviewers’ comments can be justified	1
116	Demonstrate experience or familiarity with manuscript tracking software (e.g. ScholarOne, AllenTrack, PeerTrack, BenchPress)	6
117	Demonstrate aptitude in using technology (computers, Internet, e-mail, Manuscript Submission Systems) to perform his or her editorial duties)	12
118	Possess a degree in medical editing or be trained as a journal editor	2
119	Demonstrate the ability to write editorials	2
120	Demonstrate working knowledge of the language in which the journal is published	6
121	Demonstrate skills in speed reading, skim reading, and critical reading	2
122	Demonstrate an aptitude for reading widely, deeply, and continually	3
123	Demonstrate experience and/or training in medical journal writing	11
124	Demonstrate understanding of the parts, purposes, and characteristics of tables, charts, graphs, and images	12
125	Demonstrate familiarity with scientific units, numerals, symbols, and nomenclature	17
126	Demonstrate familiarity with the presentation of data and data presentation problems	3
127	Demonstrate familiarity with the basic concepts of statistics	12
128	Demonstrate knowledge of literature reviews	1
129	Demonstrate familiarity with the principles of scientific investigation	3
130	Demonstrate familiarity with types of evidence	2
131	Demonstrate familiarity with scientific referencing	4
132	Demonstrate familiarity with clinical research design	6
133	Demonstrate knowledge of types of manuscripts	9
134	Be working towards a deeper understanding of multiple research epistemologies	1
135	Assist non-native speakers in dealing with language issues	7
VI. Ethics and integrity		
136	Demonstrate knowledge of issues around registration (i.e. trials, systematic reviews, protocols)	4
137	Demonstrate knowledge of and adherence to the principles of editorial independence	25
138	Demonstrate expertise in ensuring the ethical integrity of publications	33
139	Identify and address allegations of fraud or plagiarism	9
140	Demonstrate understanding of privacy, confidentiality, and anonymity issues	13
141	Identify and address issues related to conflicts of interest	34
142	Identify and address issues related to industry-sponsored research	6
143	Separate decision-making from commercial considerations	3
144	Demonstrate knowledge of the ethical approval process for research involving humans and animals	7
145	Ensure the respect and privacy of patients described in clinical studies	7
146	Safeguard the rights of study participants and animals	9
147	Demonstrate understanding of issues related to dual-use research (research with multiple purposes or applications)	2
148	Identify and apply appropriate reporting guidelines	5
149	Guarantee access to, and long term preservation of, the published information	3
150	Encourage debate on important topics related to the journal	2
151	Promote higher standards of medical journalism	1
152	Identify and work to avoid publication bias	3
153	Demonstrate knowledge of COPE resources for editors, authors, and peer reviewers	5
154	Demonstrate knowledge of copyright issues	2
155	Demonstrate knowledge regarding problems with multiple publications (e.g. salami, duplicate, redundant)	9
156	Identify and address incongruities and bias in manuscripts	1
157	Recommend publication of papers that meet standards of scientific rigor	2
158	Identify and address issues related to image manipulation	2
VII. Qualities and characteristics of editors		
159	Demonstrate experience and broad knowledge of the field(s) covered by the journal and of the people working in those fields	19
160	Demonstrate the ability to work in a team	14
161	Delegate/divide the workload	4
162	Communicate clearly with others	16
163	Effectively summarize manuscripts in fields outside your experience	1
164	Possess a Doctorate or Master’s Degree in related content area	8
165	Demonstrate an academic education that includes science training or experience in a research environment	17
166	Demonstrate experience and aptitude in conflict resolution	6
167	Demonstrate excellent organizational, project, and time management skills, including the ability to work under considerable time pressure	20
168	Maintain part time professional practice	2
169	Maintain membership in learned societies and editing-related associations	5
170	Be recognized as a distinguished scholar in one’s field	8
171	Maintain an active research portfolio/is employed in a research-oriented university or institute	5
172	Demonstrate past experience on an editorial board	1
173	Demonstrates competence as a practitioner in their field	2
174	Demonstrate strong interpersonal skills	5
175	Demonstrate good analytical skills	6
176	Demonstrate effective critical appraisal skills	4
177	Demonstrate the ability to achieve consensus among opinionated scientists	1
178	Demonstrate leadership skills	20
179	Demonstrate political and public relations sense	3
180	Demonstrate self-motivation	5
181	Demonstrate enthusiasm	4
182	Demonstrate tolerance and persistence	5
183	Demonstrate boldness	6
184	Demonstrate independent thinking	4
185	Maintain visibility and respect among peers and in the larger scientific community	4
186	Maintain rigid criteria	1
187	Demonstrate the ability to perpetuate or challenge master narratives	1
188	Exercise convictions with a positive attitude	1
189	Demonstrate a willingness to reconsider decisions	8
190	Demonstrate practicality	3
191	Demonstrate decisiveness	4
192	Demonstrate personal interest in medical ‘journalology’ or ‘editology’	3
193	Demonstrate an enjoyment of learning and a questioning mind	3
194	Demonstrate the desire to advance their field of study	15
195	Have access to a good academic network or have the potential to grow one	2
196	Demonstrate patience when dealing with authors and reviewers	4
197	Demonstrate knowledge of processes related to the editorial board	10
198	Respond promptly to complaints	7
199	Act with integrity and accountability	39
200	Engage with social media to reach out beyond the usual specialist audiences	22
201	Demonstrate knowledge of the parts, purposes, and characteristics of manuscripts	3
202	Demonstrate knowledge of open access models	2
Other potential competencies		
203	Statements related specifically to the Editor-in-Chief position ^d^	68

^a^ The order in which the statements are presented is purely for purposes of organization and is not intended to convey any type of ranking

^c^ Corresponds to the Item(s) columns in Tables 1 and 2

^b^ Number of extracted competency-related statements across all data sources in the scoping review

^d^ This item contains all statements pertaining only to potential competencies of Editors-in-Chief. Despite these potential competencies not being directly relevant to this scoping review, we nevertheless wanted to account for them in our results as they did fit our inclusion criteria

## Discussion

This scoping review identified 225 relevant publications, spanning more than 60 years, and involving authors from more than 20 countries. It produced a comprehensive list of possible competency-related statements for the scientific editor position within biomedical journals. This categorized list of statements will be used in a subsequent Delphi exercise aiming to ask a broad spectrum of scientific editors of biomedical journals to rate the importance of each statement in relation to performing their duties as a scientific editor. This data will then help inform a consensus meeting in which a select group of editors will collaborate to outline a set of core competencies.

Despite our finding that the competencies of editors have been discussed in the published literature since the mid-1950s, a recent systematic review [17] found no comparative studies of the effectiveness of training for editors. This is concerning, given the gate-keeping role that scientific editors play as guardians of the scientific record [23]. Trends in the number of publications annually would seem to indicate that, while the overall number of publications in this area has grown since the topic first emerged in the literature, interest peaked around 2011 and is now waning. The trend, however, is more reflective of editorial-type articles in journals, which have declined since 2011, while the number of research-based publications has remained relatively stable (yet sparse) during the same timeframe.

One possibility for the decline in total publications is that organizations may believe these issues have already been identified and they are working on training materials for them rather than publishing more research or commentaries about them in medical journals. A possibility for the continued lack of research in this area may be that the focus of many major funding agencies is to fund by disease (e.g. heart, kidney, cancer, diabetes). As journal editing, and the field of journalology more broadly, is a domain that spans across the spectrum of research on diseases, it is often difficult to find appropriate funding opportunities and even more difficult to convince specific disease-based funding agencies that the research merits their funding investment.

A major challenge that we anticipated as part of this scoping review was that a large proportion of the evidence may not be in the traditional research-based literature. For this reason, we placed a heavy emphasis on extensively searching the grey and non-research-based literature by including two related environmental scans as part of the larger program on core competencies for scientific editors of biomedical journals. However, it is still possible that we may not have captured every aspect of a biomedical editor’s work in our searches. In particular, some of the more tacit (e.g. difficult to describe) aspects of the work of editors may simply not be documented, or may reside in documents that may not necessarily be found in a database or Google search (e.g. resources residing behind membership or password-protected webpages, paywalls). We expect that any critical missing items will be brought forth in the training needs assessment, the Delphi, or the consensus meeting. Additionally, because of the subjective nature of this type of data extraction, it is possible that some competency-related statements were missed within the included publications. However, we feel that the likelihood of this was reduced as several members of the research team are editors. Finally, due to the broad inclusion criteria and the decision to preserve the wording used by authors to describe potential competencies as much as possible, it is likely that some items may not actually be competencies per se, but may instead describe tasks, behaviors, and knowledge related to competencies. However, these items are still useful in describing important aspects of editors’ work and will therefore contribute valuable information for the development of core competencies.

With the large number of competency statements and our desire to create a manageable list for use downstream in our program of research, our efforts to remove redundant and overlapping items in order to streamline the list may also have led to the elimination of some nuances between items that were subtly different from one another. While we implemented measures to ensure consistency in our methods (i.e. piloted forms, duplication of the classification exercise), ultimately there is a degree of interpretation and selectivity embedded in this process. Thus, our list of possible competencies may not include all of the competencies of biomedical editors. As noted above, the next phases of this project are designed to elicit any missing items.

Another limitation is that for feasibility purposes we only considered English and French articles, which raises the possibility that relevant information published in another language was missed. Similarly, the databases searched may not have included some journals from outside fiscally resourced countries.

## Conclusion

To our knowledge, this scoping review is the first attempt to systematically identify possible competencies of editors. On its own, the review will serve to inform readers on the extent and nature of existing literature in this area, as well as the breadth of skills, abilities, tasks, knowledge, and training that may be necessary to fulfill the position of scientific editor at a biomedical journal. More importantly, the review is part of a larger program to develop a minimum set of core competencies for scientific editors of biomedical journals.

The purpose of the 203 competency-related statements generated here is to be the central tool used for a Delphi exercise involving scientific editors of biomedical journals from around the world. Subsequently, these statements will serve to stimulate discussion at a consensus meeting in which the goal will be for relevant stakeholders to agree upon a minimum set of core competencies for scientific editors of biomedical journals. This evidence-based approach will ultimately lay the groundwork for the development of specific competency-based training and certification for scientific editors of biomedical journals [5]. The development of core competencies and subsequent training represent critical steps toward ensuring that the publication of biomedical research truly represents a hallmark of quality and trustworthiness, both within and beyond the research community.

## Additional files

Additional file 1:
**Search strategies.** (DOCX 23 kb) Additional file 2:
**Non-research-based publications.** (DOCX 49 kb)

## Abbreviations

COPE: Committee on Publication Ethics
WAME: World Association of Medical Editors
