# Supplementary material for: A scoping review of competencies for scientific editors of biomedical journals
Source: BMC Med. 2016 Feb 2;14:16. doi: 10.1186/s12916-016-0561-2 (PMC4739383; doi:10.1186/s12916-016-0561-2)
Supplement: Additional file 1: — Search strategies. (DOCX 23 kb) [file 12916_2016_561_MOESM1_ESM.docx]

**Additional file 1**

**Scoping Review of Core Competencies for Scientific Editors of Biomedical Journals**

Database: Embase Classic+Embase <1947 to 2014 November 07>, Ovid MEDLINE(R) In-Process & Other Non-Indexed Citations and Ovid MEDLINE(R) <1946 to Present>, PsycINFO <1806 to November Week 1 2014> Search Strategy:

--------------------------------------------------------------------------------

1 ((editing or editor or editors) adj5 (abilit* or aptitude* or capabilit* or capacit* or competen* or criteri* or educat* or effectiv* or expertise or integrit* or knowledg* or proficien* or qualifi* or qualify or responsibilit* or role or roles or skill or skills or standard or standards or talent* or task or tasks or training)).tw. (3854)

2 editor/ use emczd (4610)

3 professional competence/ use emczd (24501)

4 exp professional standard/ use emczd (323914)

5 responsibility/ use emczd (26196)

6 skill/ use emczd (46686)

7 training/ use emczd (66894)

8 or/3-7 (461256)

9 2 and 8 (942)

10 1 or 9 (4755)

11 remove duplicates from 10 (3664) [TOTAL UNIQUE HITS]

12 11 use prmz (1063) [MEDLINE UNIQUE HITS]

13 11 use emczd (1286) [EMBASE UNIQUE HITS]

14 11 not (12 or 13) (1315) [PSYCINFO UNIQUE HITS]

***************************

**Cochrane Library**

Search Name: Skills - Scientific Editors

Date Run: 09/11/14 21:39:40.953

Description: OHRI (JG/DM) - 2014 Nov 9

ID Search Hits

#1 ((editing or editor or editors) near/5 (abilit* or aptitude* or capabilit* or capacit* or competen* or criteri* or educat* or effectiv* or expertise or integrit* or knowledg* or proficien* or qualifi* or qualify or responsibilit* or role or roles or skill or skills or standard or standards or talent* or task or tasks or training)):ti,ab,kw 78

DSR – 3

CENTRAL – 30

Methods – 45

**CINAHL**

| **#** | **Query** | **Limiters/Expanders** | **Last Run Via** | **Results** |
| --- | --- | --- | --- | --- |
| S9 | S7 OR S8 | Expanders - Apply related words  Search modes - Boolean/Phrase | Interface - EBSCOhost Research Databases  Search Screen - Advanced Search  Database - CINAHL | 602 |
| S8 | TI ( (editing or editor or editors) N5 (abilit* or aptitude* or capabilit* or capacit* or competen* or criteri* or educat* or effectiv* or expertise or integrit* or knowledg* or proficien* or qualifi* or qualify or responsibilit* or role or roles or skill or skills or standard or standards or talent* or task or tasks or training) ) OR AB ( (editing or editor or editors) N5 (abilit* or aptitude* or capabilit* or capacit* or competen* or criteri* or educat* or effectiv* or expertise or integrit* or knowledg* or proficien* or qualifi* or qualify or responsibilit* or role or roles or skill or skills or standard or standards or talent* or task or tasks or training) ) | Expanders - Apply related words  Search modes - Boolean/Phrase | Interface - EBSCOhost Research Databases  Search Screen - Advanced Search  Database - CINAHL | 524 |
| S7 | S5 AND S6 | Expanders - Apply related words  Search modes - Boolean/Phrase | Interface - EBSCOhost Research Databases  Search Screen - Advanced Search  Database - CINAHL | 83 |
| S6 | S3 OR S4 | Expanders - Apply related words  Search modes - Boolean/Phrase | Interface - EBSCOhost Research Databases  Search Screen - Advanced Search  Database - CINAHL | 93,061 |
| S5 | S1 OR S2 | Expanders - Apply related words  Search modes - Boolean/Phrase | Interface - EBSCOhost Research Databases  Search Screen - Advanced Search  Database - CINAHL | 6,422 |
| S4 | (MH "Professional Role+") | Expanders - Apply related words  Search modes - Boolean/Phrase | Interface - EBSCOhost Research Databases  Search Screen - Advanced Search  Database - CINAHL | 62,550 |
| S3 | (MH "Professional Competence+") | Expanders - Apply related words  Search modes - Boolean/Phrase | Interface - EBSCOhost Research Databases  Search Screen - Advanced Search  Database - CINAHL | 33,920 |
| S2 | (MH "Edit and Review") | Expanders - Apply related words  Search modes - Boolean/Phrase | Interface - EBSCOhost Research Databases  Search Screen - Advanced Search  Database - CINAHL | 1,665 |
| S1 | (MH "Editors+") | Expanders - Apply related words  Search modes - Boolean/Phrase | Interface - EBSCOhost Research Databases  Search Screen - Advanced Search  Database - CINAHL | 5,033 |

**ERIC**

**2014 Nov 10**

**ti(((editing OR editor OR editors) NEAR/5 (ability* OR aptitude* OR capabilityy* OR capacity* OR competent* OR criteria* OR educate* OR effective* OR expertise OR integrity* OR knowledge* OR proficient* OR qualify* OR qualify OR responsibility* OR role OR roles OR skill OR skills OR standard OR standards OR talent* OR task OR tasks OR training))) OR ab(((editing OR editor OR editors) NEAR/5 (ability* OR aptitude* OR capabilityy* OR capacity* OR competent* OR criteria* OR educate* OR effective* OR expertise OR integrity* OR knowledge* OR proficient* OR qualify* OR qualify OR responsibility* OR role OR roles OR skill OR skills OR standard OR standards OR talent* OR task OR tasks OR training)))**

***2501 hits***

**Search Terms and Results for Core Competencies for Editors Environmental Scan (Google Search)**

|  | **Scientific Editor** | **Journal Editor** |
| --- | --- | --- |
| **Knowledge** | 2 | 0 |
| **Skill** | 5 | 11 |
| **Abilities** | 0 | 0 |
| **Aptitude** | 0 | 0 |
| **Capabilities** | 0 | 0 |
| **Expertise** | 0 | 0 |
| **Proficiency** | 0 | 1 |
| **Capacities** | 0 | 0 |
| **Education** | 0 | 0 |
| **Training** | 4 | 1 |
| **Learning** | 0 | 0 |
| **Competence** | 1 | 0 |
| **Criteria** | 0 | 0 |
| **Effective** | 1 | 0 |
| **Integrity** | 0 | 1 |
| **Qualification** | 0 | 3 |
| **Role** | 3 | 2 |
| **Standards** | 1 | 0 |
| **Responsibilities** | 7 | 3 |
| **Task** | 2 | 0 |

*The number in each cell represents the number of

documents retrieved containing competency-related

statements that were included in the review

**Search Terms for Training in Journalology Environmental Scan (Google Search)**

|  | **Author** | | **Manuscript** | | **Publisher** | | **"peer review"** | | **Journal** | | **"scientific writing"** | | **Editor** | | **"research report"** | | **Journalog*** | | **Journalolog*** | |
| --- | --- | --- | --- | --- | --- | --- | --- | --- | --- | --- | --- | --- | --- | --- | --- | --- | --- | --- | --- | --- |
|  | medical | health | medical | health | medical | health | medical | health | medical | health | medical | health | medical | health | medical | health | medical | health | medical | health |
| **Train** |  |  |  |  |  |  |  |  |  |  |  |  |  |  |  |  |  |  |  |  |
| **Educat*** |  |  |  |  |  |  |  |  |  |  |  |  |  |  |  |  |  |  |  |  |
| **Course** |  |  |  |  |  |  |  |  |  |  |  |  |  |  |  |  |  |  |  |  |
| **Curriculum** |  |  |  |  |  |  |  |  |  |  |  |  |  |  |  |  |  |  |  |  |
| **Teach** |  |  |  |  |  |  |  |  |  |  |  |  |  |  |  |  |  |  |  |  |
| **Learn** |  |  |  |  |  |  |  |  |  |  |  |  |  |  |  |  |  |  |  |  |
| **Skill** |  |  |  |  |  |  |  |  |  |  |  |  |  |  |  |  |  |  |  |  |
| **Workshop** |  |  |  |  |  |  |  |  |  |  |  |  |  |  |  |  |  |  |  |  |
| **Class** |  |  |  |  |  |  |  |  |  |  |  |  |  |  |  |  |  |  |  |  |
| **Program** |  |  |  |  |  |  |  |  |  |  |  |  |  |  |  |  |  |  |  |  |
| **Instruct** |  |  |  |  |  |  |  |  |  |  |  |  |  |  |  |  |  |  |  |  |
| **Fellowship** |  |  |  |  |  |  |  |  |  |  |  |  |  |  |  |  |  |  |  |  |
| **Internship** |  |  |  |  |  |  |  |  |  |  |  |  |  |  |  |  |  |  |  |  |
| **"quality improvement"** |  |  |  |  |  |  |  |  |  |  |  |  |  |  |  |  |  |  |  |  |
