# Supplementary material for: A scoping review of competencies for scientific editors of biomedical journals
Source: BMC Med. 2016 Feb 2;14:16. doi: 10.1186/s12916-016-0561-2 (PMC4739383; doi:10.1186/s12916-016-0561-2)
Supplement: Additional file 2: — Non-research-based publications. (DOCX 49 kb) [file 12916_2016_561_MOESM2_ESM.docx]

**Additional file 2**

**Non Research-Based Publications**

| **Author** | **Affiliation** | **Country** | **Position** | | **Journal** | **Year** | **Design** | **Funding** | **#^†^** | **Item(s)‡** |
| --- | --- | --- | --- | --- | --- | --- | --- | --- | --- | --- |
| Aguinis, H | Indiana University | USA | N/R | | Management and Organization Review | 2011 | Editorial | N/R | 5 | ^8(2), 35, 85, 200^ |
| Akhtar, J | Nat Inst of Child Health Karachi, Sindh-Pakistan. | Pakistan | Other editor | | J of Liaquat U of Med & Health Sciences | 2012 | Editorial | N/R | 6 | ^79, 98, 117, 127, 132, 157^ |
| Al Aboud, K | None listed | Saudi  Arabia | N/R | | Acta Dermatovenerol | 2005 | Editorial | N/R | 13 | ^8(2), 47, 79, 83, 95, 111, 155, 166, 194, 198, 200(2)^ |
| Albert, D | University of Wisconsin Medical School | USA | N/R | | JAMA Ophthalmology | 2005 | Editorial | EICs of Am J Ophthalmol, Arch Ophthalmol & Ophthalmol | 3 | ^25, 72, 141^ |
| Alfonso, F | Revista Española de Cardiología | Spain | Advisory Board | | Netherlands Heart Journal, | 2010 | Editorial | N/R | 6 | ^25, 26(2), 99, 141, 152^ |
| Alfonso, F | Revista Española de Cardiología | Spain | N/R | | Revista Española de Cardiología | 2008 | Editorial | N/R | 0 | ^N/A^ |
| Begley, C. G. | TetraLogic Pharmaceuticals | USA | N/R | | Pigment Cell & Melanoma Research | 2013 | Editorial | N/R | 0 | ^N/A^ |
| Berquist, T | Am Journalism Review | USA | EIC | | Am Journalism Review | 2000 | Editorial | N/R | 7 | ^35, 72, 91, 110, 189, 199, 200^ |
| Bjorneboe, J | Tidsskrift for Den norske laegeforening | France | N/R | | Medical Bulletin | 1954 | Editorial | N/R | 0 | ^N/A^ |
| Blancett, S | U of South Florida College of Nursing | USA | N/R | | Nursing Admin Quart | 1997 | Editorial | N/R | 15 | ^10, 34, 40, 45, 57(3), 72(3), 85, 91, 95, 108, 200^ |
| Bonnet, F | Hôpitaux de Paris, hôpital Tenon, université Pierre-et-Marie Curie | France | N/R | | La Presse Médicale | 2012 | Editorial | N/R | 1 | ^20^ |
| Bosch, F | Pompeu Fabra University | Spain | N/R | | Basic & Clin Pharma & Toxicology | 2011 | Editorial | N/R | 0 | ^N/A^ |
| Boucek, G | American Dental Association | USA | N/R | | 1975 Dental Editors Seminar | 1975 | Editorial | ADA Council on Journalism | 8 | ^95, 119, 122, 123, 168, 178, 179, 200^ |
| Brown, K | University of Iowa | USA | Other editor | | Acad of Management Learning & Education | 2012 | Editorial | N/R | 0 | ^N/A^ |
| Bugiardini, R | Journal of Cardiovascular Medicine | Italy | EIC | | J Cardiovascular Medicine | 2011 | Editorial | N/R | 2 | ^127, 132^ |
| Burch, G.E. | Tulane University of Medicine | USA | N/R | | Journal of Laboratory and Clinical Medicine | 1954 | Editorial | N/R | 13 | ^101, 123, 142, 165, 171, 176, 188, 194(2), 199(4)^ |
| Caelleigh, A | Academic Medicine | USA | EIC | | Academic Medicine | 1993 | Editorial | N/R | 2 | ^20, 138^ |
| Chinn, C | Educational Psychologist | USA | EIC | | Educ Psychologist | 2011 | Editorial | N/R | 0 | ^N/A^ |
| Clark, J | Public Library of Science | USA | N/R | | J Gen Intern Med | 2011 | Editorial | N/R | 0 | ^N/A^ |
| Clarke, A | None given | USA | N/R | | Nursing Forum | 1975 | Editorial | N/R | 3 | ^8, 91, 95^ |
| Collier, R | CMAJ | Canada | N/R | | CMAJ | 2011 | Editorial | N/R | 15 | ^8, 69, 160, 162, 164, 170, 176, 178, 182, 183, 189, 194, 196, 199(2)^ |
| Covet, S | American Medical Writers' Association | USA | N/R | | Mississippi Valley Medical Journal | 1956 | Editorial | N/R | 9 | ^24, 95, 96(2), 111, 120, 124, 160, 165^ |
| Cowell, J | Journal of School Nursing | USA | EIC | | J School Nursing | 2011 | Editorial | N/R | 1 | ^55^ |
| Crago, | Aus & N.Z. J Fam Therapy | Australia | EIC | | Aus & N.Z. J Fam Ther | 2008 | Editorial | N/R | 11 | ^19, 111, 159, 160, 167(2), 174, 178, 184, 196(2)^ |
| Crammer, J. L. | Neuroendocrinology Letters | UK | N/R | | Neuroendocrinology Letters | 1999 | Editorial | N/R | 2 | ^72(2)^ |
| Daly, J | Aus & N.Z. J Public Health | Australia | EIC | | Aus & N.Z. J Pub Health | 2007 | Editorial | N/R | 3 | ^8, 72, 91^ |
| Daroff, R | Medicine and Law | USA | EIC | | Medicine and Law | 2007 | Editorial | N/R | 1 | ^138^ |
| De Caterina, R | Institute of Cardiology, “G. d'Annunzio” University | Italy | EIC | | Vascular Pharmacology | 2011 | Editorial | N/R | 1 | ^139^ |
| DeBakey, L | Tulane School of Medicine | USA | N/R | | Miss. Valley Med J | 1957 | Editorial | N/R | 8 | ^8, 101, 115, 123, 156, 159, 182, 190^ |
| Desai, S | Surgisphere Corporation | USA | N/R | | J Vascular Surgery | 2011 | Editorial | N/R | 6 | ^26, 85, 137, 138, 199(2)^ |
| Eden, D | Acad Management Journal | Israel | Other editor | | Acad Management J | 2004 | Editorial | N/R | 0 | ^N/A^ |
| Feinstein, A | Yale U School of Medicine | USA | Other editor | | J Clin Epi | 1988 | Editorial | N/R | 1 | ^105^ |
| Finch, P | Conestoga College | Canada | EIC | | Int J Therapeutic Massage and Bodywork | 2011 | Editorial | N/R | 5 | ^8, 26, 57, 160, 200^ |
| Fox, P | University of Texas | USA | EIC | | Human Brain Mapping | 2009 | Editorial | N/R | 6 | ^6(2), 35(2), 63, 72^ |
| Freshwater, D | J Psychiatric and Mental Health Nursing | UK | EIC | | J Psychiatric and Mental Health Nursing | 2006 | Editorial | N/R | 5 | ^6, 91, 95, 199(2)^ |
| Gallagher, A | Nursing Ethics | UK | EIC | | Nursing Ethics | 2009 | Editorial | N/R | 6 | ^8, 72, 160, 178(2), 183^ |
| Gasparyan, A | Russell's Hall Hospital | UK | N/R | | Croatian Med J | 2011a | Editorial | N/R | 8 | ^36, 50, 72, 77, 94, 95(2), 137^ |
| Gasparyan, A | Russell's Hall Hospital | UK | N/R | | Croatian Med J | 2011b | Editorial | N/R | 6 | ^35, 95(2), 123, 169(2)^ |
| Gasparyan, A | Russell's Hall Hospital | UK | Other editor | | Inflammation and Allergy-Drug Targets | 2010 | Editorial | N/R | 2 | ^118, 169^ |
| Gelenberg, A | University of Arizona Health Sciences Center | USA | N/R | | Psychopharmacology Bulletin | 1993 | Editorial | N/R | 2 | ^8, 157^ |
| George, S | Birmingham and Solihull Mental Health NHS Foundation Trust | UK | Other editor | | Psychiatric Bulletin | 2009 | Editorial | N/R | 6 | ^165(2), 171, 180, 192, 200^ |
| Gilder, S | Tidsskrifet for Den norske Laegeforening | UK | N/R | | J of the Norwegian Medical Association | 1965 | Editorial | N/R | 5 | ^79, 89, 145, 199(2)^ |
| Glick, M | American Dental Association | USA | EIC | | Journal of the American Dental Association | 2008 | Editorial | N/R | 4 | ^72, 92, 182, 199^ |
| Goldberg, D | University of Toronto | Canada | N/R | | Clinical Biochemistry | 2006 | Editorial | N/R | 3 | ^35, 194, 197^ |
| Goozner, M | University of Connecticut | USA | N/R | | Addiction | 2009 | Editorial | Beldon Fund; Richard & Rhoda Goldman Fund; Josiah Macy Jr Foundation; Nathan Cummings Foundation and the Provost Interdisciplinary Seminar Fund of the University of Pennsylvania | 3 | ^32, 141(2)^ |
| Göthlin, JH | University of Bergen | Norway | N/R | | Euro J of Radiology | 1988 | Editorial | N/R | 10 | ^8, 14, 34, 83, 88, 110, 120, 159, 183, 200^ |
| Gottlieb, L | Can J Nursing Research | Canada | EIC | | Canadian Journal of Nursing Research | 2012 | Editorial | N/R | 4 | ^34, 65, 72(2)^ |
| Gould, J | OPTions, la Crosse, WI | USA | Former editor | | J Orthopaedic and Sports Physical Therapy | 1992 | Lecture | N/R | 6 | ^8, 26, 72, 114, 200(2)^ |
| Groves, T | BMJ | UK | Other editor | | Aus & NZ J Obstetrics and Gynaecology | 2006 | Editorial | N/R | 2 | ^141, 185^ |
| Gupta, P | University College of Medical Sciences, Delhi | India | N/R | | Indian Journal of Medical Sciences | 2008 | Editorial | N/R | 0 | ^N/A^ |
| Hartley, J | School of Psychology, University of Keele | UK | Other editor | | British Journal of Educational Technology | 2012 | Editorial | N/R | 0 | ^N/A^ |
| Henley, L | University of Cape Town | South Africa | N/R | | Indian J Med Sciences | 2008 | Editorial | N/R | 2 | ^138, 148^ |
| Hitt, M | Texas A&M University | USA | N/R | | Organizational Research Methods | 2009 | Editorial | N/R | 2 | ^34, 200^ |
| Hwang, K | Inha University | Korea | Other editor | | Arch Plastic Surgery | 2013 | Editorial | N/R | 4 | ^7, 39, 104(2)^ |
| Isaacs, D | Journal of Pediatrics and Child Health | Australia | EIC | | Journal of Pediatrics and Child Health | 2014 | Editorial | N/R | 0 | ^N/A^ |
| Jabri, E | American Chemical Society | USA | N/R | | The FASEB Journal | 2015 | Editorial | N/R | 7 | ^88, 160(2), 164, 165, 175, 178^ |
| Jawaid, S | Pakistan Journal of Medical Sciences | Pakistan | Other editor | | Pakistan Journal of Medical Sciences | 2013 | Editorial | N/R | 9 | ^8(2), 30(2), 79, 107,199, 200(2)^ |
| Jawaid, S | Pakistan Journal of Medical Sciences | Pakistan | N/R | | Saudi Medical Journal | 2004 | Editorial | N/R | 3 | ^6, 195, 199^ |
| Jawaid, S | Pakistan Journal of Medical Sciences | Pakistan | Other editor | | Pakistan Journal of Medical Sciences | 2011 | Editorial | N/R | 14 | ^6, 9, 20, 33, 54, 72(3), 95, 124, 138, 140, 149, 167^ |
| Jawaid, S | Pakistan Journal of Medical Sciences | Pakistan | N/R | | The Journal of Tehran University Heart Center | 2008 | Editorial | N/R | 10 | ^6, 30, 72(3), 79, 91, 145, 199, 200^ |
| Jennings, W | University of South Florida | USA | EIC | | American Journal of Criminal Justice | 2012 | Editorial | N/R | 2 | ^85, 92^ |
| Jones, R | Department of Gynecological Oncology, National Women’s Hospital, Auckland, New Zealand | New Zealand | N/R | | Obstetrics & Gynecology | 2009 | Editorial | N/R | 0 | ^N/A^ |
| Kalra, G | Psychiatry Registrar, Northern CCU, Northwestern Mental Health | Australia | N/R | | Asian Journal of Psychiatry | 2013 | Editorial | N/R | 7 | ^6, 26, 72, 105, 110, 116, 167^ |
| Kassirer, J | Tufts University School of Medicine | USA | N/R | | Maryland Medicine | 2007 | Editorial | N/R | 4 | ^104, 137, 199(2)^ |
| Kennedy, M | American Journal of Nursing | USA | EIC | | American Journal of Nursing | 2014 | Editorial | N/R | 2 | ^2, 20^ |
| King, L | Department of Psychological Sciences, University of Missouri | USA | N/R | | Psychological Inquiry | 2012 | Editorial | N/R | 1 | ^8^ |
| Kipping, P | Hammill Institute on Disabilities | USA | N/R | | Assessment for Effective Intervention | 2008 | Editorial | N/R | 0 | ^N/A^ |
| Klionsky, D | University of Michigan; Life Sciences Institute | USA | EIC | | Autophagy | 2008 | Editorial | N/R | 0 | ^N/A^ |
| Koch, D | Journal of Cataract & Refractive Surgery | USA | EIC | | Journal of Cataract & Refractive Surgery | 2007 | Editorial | N/R | 0 | ^N/A^ |
| Lapeña, J | Journal of Otolaryngology – Head and Neck Surgery | Philippines | N/R | | Singapore Medical Journal | 2009 | Editorial | N/R | 10 | ^6(2), 72(2), 80, 104, 137, 138, 199(2)^ |
| Lee, K. | None given | Canada | N/R | | 12th Cochrane Colloquium | 2004 | Editorial | N/R | 0 | ^N/A^ |
| Liesegang, T | Mayo Clinic | USA | EIC | | American Journal of Ophthamology | 2003 | Editorial | N/R | 3 | ^26, 57, 91^ |
| Luce, M | Not given | USA | EIC | | Journal of Consumer Research | 2012 | Editorial | N/R | 1 | ^35^ |
| Mait, J | Applied Optics | USA | EIC | | Applied Optics | 2013 | Editorial | N/R | 2 | ^36, 39^ |
| Marcovitch, H | Clinical Risk | UK | Other editor | | Archives of Disease in Childhood | 2009 | Editorial | N/R | 2 | ^162, 190^ |
| Marcovitch, H | Not given | UK | N/R | | Journal of Medical Ethics | 2012 | Editorial | N/R | 2 | ^72, 138^ |
| Martin, A | Yale Child Study Center | USA | Scientific Editor | | Academic Psychiatry | 2014 | Editorial | N/R | 11 | ^8, 39, 72, 91, 95, 104, 113, 138, 161, 178, 199^ |
| Marušić, A | Department of Research in Biomedicine and Health Co-EIC, Journal of Global Health, University of Split School of Medicine, Croatia | Croatia | N/R | | Conference on Excellence in Rheumatology | 2012 | Editorial | N/R | 0 | ^N/A^ |
| Marušić, A | Department of Research in Biomedicine and Health, University of Split School of Medicine, Split, Croatia | Croatia | N/R | | Biochemia Medica | 2012 | Editorial | NR | 1 | ^138^ |
| Marušić, A | Department of Anatomy, School of Medicine, Zagreb University, Salata, Croatia | Croatia | N/R | | Medical Education | 2005 | Editorial | N/R | 4 | ^72(2), 92, 199^ |
| Marušić, A | Croatian Medical Journal and Department of Research in Biomedicine and Health, University of Split, Split, Croatia | Croatia | EIC | | Biochemia Medica | 2010 | Editorial | N/R | 14 | ^28, 61, 72(3), 79, 83, 137, 138, 158, 170, 180, 185, 199^ |
| Marušić, M | Zagreb University School of Medicine, Croatia | Croatia | EIC | | Croatian Medical Journal | 2001 | Editorial | N/R | 1 | ^8^ |
| Matocha, L | College of Nursing, University of Delaware | USA | N/R | | Marriage & Family Review | 1993 | Editorial | N/R | 6 | ^6, 7, 8, 34, 46, 167^ |
| Meltzoff, J | None listed | USA | N/R | | Journal of Aggression, Maltreatment & Trauma | 2005 | Editorial | N/R | 2 | ^72, 170^ |
| Miettinen, O | McGill University | Canada | N/R | | Canadian Medical Association Journal | 1999 | Editorial | N/R | 0 | ^N/A^ |
| Montorsi, F | Department of Urology, Universita’ Vita Salute San Raffaele | Italy | EIC | | European Urology | 2006 | Editorial | N/R | 1 | ^35^ |
| Mullen, C | The University of North Carolina at Greensboro | USA | EIC | | Mentoring & Tutoring: Partnership in Learning | 2009 | Editorial | N/R | 5 | ^8(2), 90(2), 194^ |
| None | None | Croatia | WAME | | Croatian Medical Journal | 2001 | Editorial | N/R | 4 | ^57, 72(2), 200^ |
| Pariante, C | Institute of Psychiatry, Kings College London | UK | Other type of editor | | Epidemiologia e Psichiatria Sociale | 2010 | Editorial | N/R | 14 | ^20, 68, 72(2), 73(2), 113, 117, 139,155, 178, 183, 194, 199^ |
| Patel, V | Department of Pharmacology, Smt. Nathiba Hargovandas Lakhmichand Municipal Medical College | India | EIC | | Indian Journal of Pharmacology | 2013 | Editorial | N/R | 4 | ^20, 34, 97, 105^ |
| Pitkin, R | Obstetrics & Gynecology | USA | EIC | | Obstetrics & Gynecology | 1998 | Editorial | N/R | 6 | ^6, 46, 48, 139, 141, 155^ |
| Poster, E | Journal of Child and Adolescent Psychiatric Nursing | USA | N/R | | Perspectives in Psychiatric Care | 2012 | Editorial | N/R | 4 | ^72(2), 138, 194^ |
| Povl R | None given | Denmark | N/R | | Acta Medica Scandinavica | 1988 | Editorial | N/R | 8 | ^6, 27, 68, 91, 120, 127, 132, 175^ |
| Pressley, M | University of Albany, State University of New York | USA | N/R | | Educational Psychology Review | 1994 | Editorial | N/R | 0 | ^N/A^ |
| Punjabi, P | Perfusion | UK | EIC | | Perfusion | 2010 | Editorial | N/R | 12 | ^6(2), 26, 33, 34, 35(2), 41, 72(3), 138^ |
| Punjabi, P | Perfusion | EIC | | | Perfusion | 2010 | Editorial | N/R | 0 | ^N/A^ |
| Randell-Moon, H | Macquarie University, North Ryde, Sydney | Australia | | N/R | Ethics and Education | 2011 | Editorial | N/R | 13 | ^6(2), 8, 30, 34, 35, 57, 145, 164, 178, 189, 194, 200^ |
| Ray, JG | University of Toronto | Canada | | N/R | QJM | 2002 | Editorial | N/R | 2 | ^137, 165^ |
| Rees, M | University of Medicine and Dentistry of New Jersey Women’s Centre, John Radcliffe Hospital | USA | | N/R | Maturitas | 2012 | Editorial | N/R | 1 | ^20^ |
| Relman, A | The New England Journal of Medicine | USA | | N/R | Ugeskrift for Laeger | 1990 | Editorial | N/R | 5 | ^8, 65, 145, 165, 200^ |
| Rennie, D | JAMA | USA | | N/R | JAMA | 1991 | Editorial | N/R | 0 | ^N/A^ |
| Rennie, D | JAMA | USA | | Other editor | JAMA | 1991 | Editorial | N/R | 0 | ^N/A^ |
| Reynolds, T | Department of Emergency Medicine, University of California, San Francisco | USA | | N/R | Annals of Emergency Medicine | 2010 | Editorial | N/R | 6 | ^65, 79, 95, 123, 138, 176^ |
| Rifai, N | Department of Laboratory Medicine, University-Hospital, Padova, Italy | Italy | | EIC | Clinical Chemistry and Laboratory Medicine | 2011 | Editorial | N/R | 4 | ^84, 89, 102, 178^ |
| Rifai, N | Clinical Chemistry | USA | | EIC | Clinical Chemistry | 2011 | Editorial | N/R | 2 | ^23, 72^ |
| Sambunjak, D | Zagreb University School of Medicine | Croatia | | N/R | Journal of Public Health Policy | 2006 | Editorial | N/R | 0 | ^N/A^ |
| Sanders, W. G. | Rice University | USA | | Other editor | The Academy of Management Journal | 2009 | Editorial | N/R | 0 | ^N/A^ |
| Sarr, M | Mayo Clinic and Mayo Foundation | USA | | EIC | Surgery | 2002 | Editorial | N/R | 2 | ^23, 91^ |
| Satyanarayana, K | Indian Journal of Medical Research | India | | N/R | Indian Journal of Medical Research | 2010 | Editorial | N/R | 2 | ^35, 186^ |
| Schiedermayer, D | Pritzker School of Medicine, University of Chicago | USA | | N/R | JAMA Internal Medicine | 1986 | Editorial | National Fund for Medical Education, Henry J. Kaiser Family Foundation, Andrew W. Mellon Foundation | 5 | ^6, 106, 111, 140, 199^ |
| Schifferes, J | American Medical Writers' Association | USA | | N/R | New York State Journal of Medicine | 1961 | Editorial | N/R | 13 | ^5, 35, 72, 82, 91, 95, 111, 167, 183, 184, 194, 200(2)^ |
| Schneider, B | University of California, Santa Barbara | USA | | N/R | Gender & Society | 2011 | Editorial | N/R | 2 | ^159, 167^ |
| Schneider, M | St. Tammany Parish Public Schools | USA | | Other editor | Journal of Mental Health Counseling | 2009 | Editorial | N/R | 2 | ^8, 72^ |
| Sher, K | University of Missouri—Columbia | USA | | N/R | American Psychologist | 2002 | Editorial | N/R | 0 | ^N/A^ |
| Smith, E | Canadian Journal of Cardiology | Canada | | EIC | Canadian Journal of Cardiology | 2006 | Editorial | N/R | 1 | ^137^ |
| Smoyak, S | Journal of Psychiatry & Neuroscience | USA | | EIC | Journal of Psychiatry & Neuroscience | 2011 | Editorial | N/R | 1 | ^4^ |
| Snyder, P | Louisiana State University Health Sciences Center | USA | | N/R | American Educational Research Association | 2001 | Editorial | N/R | 9 | ^3, 8, 15, 72(3), 95, 127(2)^ |
| Spitzer, W | McGill University | Canada | | Editor Emeritus | Journal of Clinical Epidemiology | 2002 | Editorial | N/R | 2 | ^33, 123^ |
| Stanley, C | None given | USA | | N/R | Educational Researcher | 2007 | Editorial | N/R | 10 | ^6, 26, 39, 44, 73, 134, 176, 187, 197, 199^ |
| Stapleton, J | Addiction | UK | | Other editor | Addiction | 2012 | Editorial | N/R | 2 | ^31, 160^ |
| Stoffers, J | Maastricht University | Netherlands | | EIC | European Journal of General Practice | 2011 | Job Description | N/R | 11 | ^26, 101, 120, 138, 148, 164, 165, 167, 173, 180, 192^ |
| Suh, S | Department of Bio-based Products, College of Natural Resources, University of Minnesota | USA | | N/R | The International Journal of Life Cycle Assessment | 2006 | Editorial | N/R | 5 | ^9, 26, 72, 199(2)^ |
| Tansella, M | Epidemiologia e Psichiatria Sociale | Italy | | EIC | Epidemiologia e Psichiatria Sociale | 2011 | Editorial | N/R | 11 | ^14, 20, 68, 72(3), 95, 102, 103, 104, 183^ |
| Tebbetts, J.B. | Not reported | USA | | N/R | Plastic and Reconstructive Surgery | 2004 | Editorial | N/R | 1 | ^194^ |
| Thomson, W | The Practitioner | UK | | N/R | The Medical Journal of Australia | 1954 | Editorial | N/R | 4 | ^72(2), 111, 179^ |
| Van Der Weyden, M | Medical Journal of Australia | Australia | | N/R | Medical Journal of Australia | 2006 | Editorial | N/R | 1 | ^137^ |
| Van Norman, G | University of Washington | USA | | N/R | Anesthesia & Analgesia | 2005 | Editorial | N/R | 2 | ^144, 146^ |
| Vosshall, L | Howard Hughes Medical Institute, Laboratory of Neurogenetics and Behavior, The Rockefeller University | USA | | Other editor | The FASEB Journal | 2012 | Editorial | N/R | 3 | ^107, 160, 191^ |
| Wachs, J | College of Nursing, East Tennessee State University | USA | | Other type of editor | American Association of Occupational Health Nurses | 2003 | Interview | N/R | 0 | ^N/A^ |
| Wager, E | Sideview, Princes Risborough | UK | | N/R | Medicine and Law | 2007 | Editorial | N/R | 2 | ^20(2)^ |
| Wager, E | Sideview, Princes Risborough | UK | | N/R | Anaesthesia | 2012 | Editorial | N/R | 1 | ^20^ |
| Yankauer, A | American Journal of Public Health | USA | | EIC | American Journal of Public Health | 1990 | Editorial | N/R | 0 | ^N/A^ |
| Zandi, I | Not Reported | USA | | N/R | Plastic and Reconstructive Surgery | 2004 | Editorial | N/R | 1 | ^199^ |
| Zeitoun, J-D | Centre hospitalier Diaconesses-Croix Saint-Simon, Hôpital Saint-Antoine | France | | N/R | La Presse Medicale | 2012 | Editorial | N/R | 0 | ^N/A^ |
| Zelen, M | Harvard School of Public Health | USA | | N/R | Journal of Clinical Oncology | 1983 | Editorial | N/R | 1 | ^72^ |
| zur Hausen, H | International Journal of Cancer | Germany | | EIC | International Journal of Cancer | 2006 | Editorial | N/R | 1 | ^141^ |
| TOTAL | | | | | | | | | 537 |  |

**†** Number of competency statements extracted from the document

**‡** Item(s): Corresponds to the item number from the list of statements in Table 3
